# Exploratory Metaviromic Analysis of the Sea-Rock Pool Mosquito *Aedes mariae* and the Water of Its Breeding Habitat

**DOI:** 10.3390/biology15120940

**Published:** 2026-06-16

**Authors:** Pamela Mancini, David Brandtner, Giulia Cordeschi, Marcello Iaconelli, Valentina Mastrantonio, Giuseppina La Rosa, Daniele Porretta

**Affiliations:** 1 National Center for Water Safety (CeNSiA), Istituto Superiore di Sanità, Viale Regina Elena 299, 00161 Roma, Italy; david.brandtner@iss.it (D.B.); marcello.iaconelli@iss.it (M.I.); giuseppina.larosa@iss.it (G.L.R.); 2 Department of Ecology and Biology, Tuscia University, Via Santa Maria in Gradi 4, 01100 Viterbo, Italy; giulia.cordeschi@unitus.it; 3 Department of Environmental Biology, Sapienza University of Rome, Piazzale Aldo Moro 5, 00185 Roma, Italy; valentina.mastrantonio@uniroma1.it (V.M.); daniele.porretta@uniroma1.it (D.P.)

**Keywords:** *Aedes mariae*, mosquito virome, metagenomics, long-read sequencing, sea-rock pool, aquatic environment, viral diversity, exploratory viromics

## Abstract

The mosquito-associated virome may modulate host biology and influence vector competence, highlighting the importance of understanding its composition. Here, a metagenomic analysis was conducted to characterize the virome of the sea-rock pool mosquito *Aedes mariae* across sexes and developmental stages, together with water from its sea-rock pool breeding site in San Felice Circeo (Italy). A total of 51 viral taxa were identified, including viruses associated with bacteria and archaea (39%), plants, algae, fungi, and protists (35%), vertebrates (8%), and invertebrates (18%), including insect-specific viruses such as *Mesoniviridae*, *Baculoviridae*, *Nudiviridae*, *Iridoviridae* and *Totiviridae*. Twenty-five percent of the taxa were shared across samples, suggesting acquisition from breeding-site water and persistence across stages during development. Interestingly, the need for host genome filtering highlights the potential sequence similarity between viral and mosquito genomes, which may reflect the presence of endogenous viral elements or historical virus–host interactions. These findings represent the first characterization of the virome of *Aedes mariae* and highlight the role of aquatic breeding sites in shaping mosquito virome. Finally, we argue the importance of adequate sequencing depth and host genome filtering to capture the diversity of the mosquito virome.

## Simple Summary

This exploratory study investigates the viral communities associated with the mosquito *Aedes mariae* and the saline water where its larvae develop along the Italian coast. Although mosquitoes naturally harbor many viruses, little is known about the viromes of species adapted to saline habitats. Using a long-read sequencing approach, we analyzed viruses present in larvae, pupae, adults, and breeding-site water. We identified 51 viral groups, including viruses associated with bacteria, plants, fungi, invertebrates, and vertebrates. Several viral groups were detected both in water and mosquito samples across developmental stages, indicating potential ecological links between the aquatic environment and the mosquito-associated virome. As an exploratory study, the analytical strategy was designed to maximize the detection of viral diversity. Overall, this work provides the first overview of the virome of *Aedes mariae* and establishes a foundation for future ecological and virological studies of mosquitoes associated with saline habitats.

## 1. Introduction

Mosquitoes (Diptera: Culicidae) are a large and diverse taxonomic group, comprising over 3600 described species distributed across temperate and tropical regions, and even beyond the Arctic Circle, with peak diversity in tropical forests [1,2,3]. The extant species are grouped into two subfamilies: Anophelinae, which includes the genus *Anopheles* (the malaria vector), and Culicinae, with 110 genera including *Aedes* and *Culex*, which are responsible for the transmission of a wide range of viral pathogens [1,4].

Through specific adaptations, mosquitoes have colonized a wide range of aquatic and terrestrial environments. Their holometabolous life cycle includes aquatic immature stages (egg, larva, pupa) and a winged adult stage. The immature stages can develop in a variety of permanent and temporary aquatic habitats such as ponds, swamps, river floodplains, or irrigation waters. Some species have also adapted to lay eggs in artificial containers, including buckets, tires, and planter trays [1,5,6]. The breeding site environment plays a crucial role in the acquisition of the mosquito microbiota [7], which can influence mosquito physiology, behavior, and vector competence [8]. Studies have shown that larvae belonging to different species from the same breeding site share more similar bacterial communities than conspecifics from different sites [9], and that part of this microbiome can persist across all developmental stages [10].

Viruses are a key component of the mosquito microbiome. Environmental exposure plays an important role in their acquisition by individual mosquitoes [11], and several studies have shown that many viruses detected in mosquitoes originate from organisms present in their surrounding environment, including plants, fungi, algae, protists, and aquatic habitats [12]. These viruses also include vertebrate-pathogenic viruses, insect-specific viruses, which replicate exclusively in arthropods, and viruses associated with symbiotic or commensal microorganisms of mosquitoes. However, despite their ecological relevance, most microbiome studies in mosquitoes have primarily focused on bacterial communities [13,14]. In recent years, the study of the mosquito virome has gained increasing attention. The application of next-generation sequencing (NGS) has led to the discovery of numerous mosquito-specific viruses that, although restricted to arthropod hosts and unable to replicate in vertebrate cells, are phylogenetically related to medically relevant arboviruses belonging to families such as *Bunyaviridae*, *Flaviviridae*, *Reoviridae*, and *Togaviridae* [15,16,17]. Several studies suggest that some pathogenic arboviruses evolved from arthropod-associated ancestors, highlighting the evolutionary relevance of insect-specific viruses [18,19]. From a public health perspective, viruses can influence vector competence. For example, insect-specific viruses (ISV) may induce “superinfection exclusion,” whereby a primary ISV infection prevents subsequent viral infections in the same host [20]. At the same time, viruses may also be relevant to host biology by affecting behavior, reproduction, and immunity [20,21,22,23,24]. Despite its ecological and public health relevance, the mosquito virome remains poorly characterized, as most studies have focused on a limited number of mosquito species that are vectors of human pathogens, such as *Aedes albopictus*, *Aedes aegypti*, *Culex pipiens*, *Culex quinquefasciatus*, and *Anopheles gambiae*. Likewise, the mechanisms underlying the acquisition and transmission of these viruses remain poorly understood. Although vertical transmission has been documented [18], the role of environmental acquisition -particularly from aquatic developmental sites—has so far received limited attention. Furthermore, although some mosquito species develop in brackish or saline waters, these environments have received far less attention than freshwater habitats. Characterizing the virome of mosquito species developing in these environments is of interest, also considering the ongoing salinization processes and the potential adaptation of some freshwater species to brackish conditions.

In this study, we focused on the sea-rock pool mosquito *Aedes mariae*. This species is distributed along the coasts of the Mediterranean basin, where it develops in the sea-rock pools [6,25,26]. These habitats are characterized by extreme and highly variable salinity conditions, ranging from near freshwater to hypersaline levels exceeding seawater salinity (up to 87.5 ppt), which limits the number of mosquito species able to colonize them worldwide [27,28].

Mosquitoes adapted to highly saline or brackish breeding habitats represent a relatively small ecological group that includes only a few species complexes and taxa, among them the *Aedes mariae* complex (*Aedes mariae*, *Aedes zammitii*, and *Aedes phoeniciae*) [25,29,30,31] along the Mediterranean coasts, *Opifex fuscus* in New Zealand, *Aedes australis* (*Haledes australis*) in Australia, and *Aedes togoi* in Canada [25]. Other species developing in brackish waters in the Eurasian region include *Aedes dorsalis*, *Aedes detritus* [30,32,33], and *Ochlerotatus caspius* [30].

Despite their adaptation to environmentally extreme habitats characterized by strong salinity fluctuations, information on the viromes associated with these mosquitoes remains extremely limited. To the best of our knowledge, studies investigating the viromes of mosquitoes associated with saline environments are currently limited to *Ochlerotatus caspius*, for which only the RNA virome has been characterized [34]. Consequently, the virome of *Aedes mariae* and, more generally, information on the viromes of species belonging to the *Aedes mariae* complex remained unexplored prior to the present study.

Our specific aims were: (1) to characterize the virome of the species across development; (2) to investigate the role of breeding site water in the mosquito virome acquisition during development. For this aim, we applied a third-generation long-read metagenomic approach (Oxford Nanopore Technologies, ONT) to both a rock-pool water sample and to *Aedes mariae* individuals (larvae, pupae, and adult males and females), collected along the Tyrrhenian Sea coast (San Felice Circeo, Central Italy).

## 2. Materials and Methods

### 2.1. Sampling and Nucleic Acid Extraction

#### 2.1.1. Water Sampling and Processing

Sampling was conducted in October 2022 along the Tyrrhenian Sea coast, in the municipality of San Felice Circeo, Central Italy (41°13′20.78″ N, 13°04′07.51″ E). A total of 20 L of rock-pool water was collected from a shallow sea-rock pool (maximum depth < 0.5 m) for the analysis of the viral community. Water was collected in sterile containers to prevent external contamination, transported to the laboratory at a controlled temperature of +4 °C and processed on the same day to ensure sample integrity. All analyses were conducted at the laboratory of the Istituto Superiore di Sanità. The water sample was subjected to filtration and concentration following the EPA Method 1615 protocol [35]. The process involved filtration using sterile electropositive filters 3M Zeta Plus 1MDS (3M, St. Paul, MN, USA). After filtration, the filter was eluted with 50 mL of 3% beef extract (pH 9.5), placed in a sterile container and kept under gentle agitation (300× *g*) for 30 min. The resulting beef extract eluate was then subjected to a secondary concentration step through organic flocculation. The eluate was transferred into a beaker and placed on a magnetic stirrer, agitated at a speed sufficient to create a vortex. The pH was adjusted to 3.5 ± 0.1 by adding 1.2 M HCl. Subsequently, the beef extract suspension was centrifuged at 2500× *g* for 15 min at 4 °C, and the supernatant was discarded. The pellet containing viruses was then resuspended in 10 mL of 0.15 M sodium phosphate buffer (Na_3_PO_4_, pH 9.0); of these, 5 mL were stored at −20 °C as a backup sample, while the remaining 5 mL were used for extraction. Nucleic acids were extracted using the MiniMag semi-automatic platform (bioMerieux, Paris, France) following the manufacturer’s instructions. The eluted nucleic acids were purified using ZYMO OneStep PCR Inhibitor Removal Kit (Zymo Research, Irvine, CA, USA) and immediately stored at −80 °C until further analysis. To monitor potential contamination arising during sample processing, an extraction blank composed of molecular biology-grade water (DNase-, RNase-, and protease-free) and lacking biological material was included as a negative control. The blank underwent all nucleic acid extraction procedures in parallel with the mosquito samples using the same reagents and experimental workflow.

#### 2.1.2. Mosquito Individuals Sampling and Processing

*Aedes mariae* larvae and pupae were sampled from the same rock pool and at the same time as the water sample, and were placed in plastic trays filled with water from the pool of origin before being brought to the laboratory. A subset of larvae and pupae was placed at −80 °C until genetic analyses, while the remaining specimens were left in the water until adulthood. Then the newly emerged adult males and females were kept at −80 °C until genetic analyses. This approach ensures that the analysis of the adult virome is maintained across development stages, rather than viruses acquired at the adult stage from the environment.

All individuals analysed were identified to species and sex using the morphological keys of Becker et al. 2020 [1]. Five *Aedes mariae* individuals for each stage and sex (L4 instar larvae, male and female pupae, male and female adults) were pooled and analyzed using metagenomic analysis. Before proceeding with nucleic acid extraction, mosquito specimens were treated with 1% hypochlorite for 30 s to remove contaminating microorganisms and rinsed with sterile Milli-Q^®^ water (Milli-Q^®^ Integral System, Merck Millipore, Burlington, MA, USA) for 30 s. The specimens were then placed in a tube to which 220 μL of Phosphate-Buffered Saline was added and manually homogenized using SP Bel-Art^®^ Proculture Plastic Pestles for Micro-Tube Homogenizer System (SP Bel-Art, Wayne, NJ, USA). To remove debris from the specimens, the sample was centrifuged at 16,000× *g* for 5 min at 4 °C, and the supernatant (approximately 200 μL) was recovered. Nucleic acids were then extracted using the ZymoBIOMICS DNA/RNA Miniprep Kit (Zymo Research, Irvine, CA, USA), following the manufacturer’s instructions. This kit simultaneously isolates both DNA and RNA in a single nucleic acid extraction. The eluted nucleic acids were immediately stored at −80 °C until library preparation. To monitor potential contamination arising during sample processing, an extraction blank composed of molecular biology-grade water (DNase-, RNase-, and protease-free) and lacking biological material was included as a negative control. The blank underwent all nucleic acid extraction procedures in parallel with the mosquito samples using the same reagents and experimental workflow.

### 2.2. Double-Stranded Complementary DNA (cDNA) Synthesis and Sequence-Independent Single Primer Amplification (SISPA)

Metagenomic cDNA from water and mosquito samples was generated using the Sequence-Independent Single Primer Amplification (SISPA) technique, following the protocol described by Moreno & O’Connor [36]. Reverse transcription and second-strand cDNA synthesis were performed during the initial phase (SISPA A: Reverse Transcription and 2nd strand cDNA synthesis—Primer A Addition A), in which RNA was reverse transcribed using the SuperScript IV First-Strand Synthesis System (Thermo Fisher Scientific™, Waltham, MA, USA) in combination with Primer A (5′-GTTTCCCACTGGAGGATA-N9-3′). Subsequently, second-strand synthesis was carried out using Sequenase DNA polymerase (Affymetrix, Santa Clara, CA, USA). The cDNA amplification step (SISPA B: PCR Amplification of Randomly Primed cDNA—cDNA Amplification) was then performed using AccuTaq LA DNA polymerase (Sigma-Aldrich, St. Louis, MO, USA) and 1 μL of Primer B (5′-GTTTCCCACTGGAGGATA-3′, 100 pmol/μL). The modified PCR conditions were as follows: initial denaturation at 98 °C for 30 s, followed by 30 cycles of 94 °C for 15 s, 50 °C for 20 s, and 68 °C for 5 min, with a final extension at 68 °C for 10 min. Because total nucleic acids were extracted, the SISPA workflow enabled the simultaneous analysis of viral DNA genomes and RNA virus genomes converted into cDNA. Consequently, the final sequencing library represented both DNA viruses and RNA viruses. In addition, because no enzymatic treatment was performed prior to SISPA amplification to selectively remove either DNA or RNA molecules, the resulting library may also include viral transcripts. Therefore, the resulting data were interpreted as a broad representation of the viral genetic repertoire present in the samples rather than exclusively viral genomic sequences. The amplified DNA and cDNA were then purified and size-selected using MagSi-NGSPREP Plus (Magtivio, Nuth, The Netherlands), applying a bead-to-sample ratio of 0.55×, following the manufacturer’s instructions. This ratio enables the retention of DNA fragments of approximately ≥ 500 bp, while shorter fragments are effectively removed. Following SISPA amplification and purification, DNA concentration and fragment size distribution were assessed using the Qubit™ 1X dsDNA High Sensitivity (HS) Assay Kit and Qubit™ 4 Fluorometer (Thermo Fisher Scientific™, Waltham, MA, USA), and the QIAxcel Advanced System (QIAGEN, Hilden, Germany), respectively. The extraction blanks processed in parallel with the water and mosquito samples and subjected to the same workflow yielded similarly low amounts of DNA and exclusively short DNA fragments (length below 100 bp). Consequently, both negative controls were excluded prior to library preparation and were not included in sequencing.

### 2.3. Library Preparation and Sequencing

A total of six libraries were prepared, each consisting of a pool of five individuals, corresponding to six sequenced samples: rock pool water (Dataset 1) [37], *Aedes mariae* L4 larvae (Dataset 2) [37], female pupae (Dataset 3) [37], male pupae (Dataset 4) [37], adult females (Dataset 5) [37], adult males (Dataset 6) [37]. A schematic overview of the complete workflow, including library preparation, sequencing and downstream bioinformatic analyses, is provided in Supplementary Figure S1. Before proceeding with library preparation, the 260/280 and 260/230 nucleic acid quality ratios were spectrophotometrically analyzed for each of the samples using NanoDrop™ Spectrophotometers (Thermo Fisher Scientific™, Waltham, MA, USA) and the molecular weight of the nucleic acids was analyzed using the Qubit™ 4 Fluorometer (Thermo Fisher Scientific™, Waltham, MA, USA). The metagenomic analysis was conducted using the third-generation long-read sequencing technology from Oxford Nanopore Technologies (ONT) (Oxford, UK). Library preparation was carried out using the Ligation Sequencing Kit 9 (SQK-LSK109) along with the Native Barcoding Expansions (EXP-NBD104 and EXP-NBD114), following the manufacturer’s protocol (NBE_9065_v109_revAP_14Aug2019). Sequencing was performed using FLO-MIN106 (R9.4.1) flow cells (Oxford Nanopore Technologies, Oxford, UK) with the Nanopore MinION Mk1B (Oxford Nanopore Technologies, Oxford, UK) sequencer under MinKNOW v22.12.7 with Guppy v6.4.6 basecaller using the High Accurate Model for basecalling (reads quality filter set to Phred score > 9).

### 2.4. Bioinformatic Analysis

The raw reads generated from MinION sequencing consist of multiple folders containing compressed fastq.gz files. Prior to analysis, all files were concatenated into a single fastq.gz file. For this purpose, the Concatenate datasets tail-to-head (cat) (Galaxy Version 9.3+galaxy1) [38] tool was used. To ensure the high quality of the data used for analyses, a preliminary preprocessing phase was conducted. Adapter removal from raw reads generated by the Oxford Nanopore platform was performed using Porechop (Galaxy Version 0.2.4+galaxy0) [39] with default settings, resulting in a significant reduction in data noise, improved sequence assembly accuracy, and a high overall quality of the preprocessed reads. For read filtering based on length and quality criteria, Filtlong Filtering long reads by quality (Galaxy Version 0.2.1+galaxy0) [40] was used with default parameters, except for the “Min. length” parameter, which was set to 400 bp to exclude all sequences shorter than this threshold. Subsequently, fastp- fast all-in-one preprocessing for FASTQ files (Galaxy Version 0.23.2+galaxy0) [41] was employed with the tool’s default settings to filter out reads with low *Phred* (Q) scores. Finally, the quality of the reads was assessed prior to metagenomic analysis using FastQC Read Quality reports (Galaxy Version 0.74+galaxy0) [42]. Following this preprocessing phase, and prior to taxonomic classification, a host genome depletion step was performed to reduce host-derived background and improve the specificity of downstream metagenomic analyses. Viral integrations have been documented in different *Aedes* mosquito species [43,44]. Therefore, before proceeding with the virome analysis, a host genome depletion step was performed to reduce host-derived background prior to metagenomic profiling. Sequencing reads were aligned against a mosquito reference genome, and reads showing reliable host alignments were removed. As no reference genome is currently available for *Aedes mariae*, the chromosome-level *Aedes aegypti* AaegL5.0 assembly was selected as the most complete and well-annotated chromosome-scale reference genome currently available within the genus *Aedes*. Although *Aedes aegypti* and *Aedes mariae* belong to different evolutionary lineages within the genus, *Aedes aegypti* currently represents the most comprehensive and extensively curated genomic resource available, making it a suitable reference for the removal of conserved host-derived sequences in the absence of a species-specific genome. The host reference was downloaded from NCBI (assembly accession GCF_002204515.2, AaegL5.0) and the genomic FASTA file (GCF_002204515.2_AaegL5.0_genomic.fna) was used to build a minimap2 index (.mmi). Reads were aligned to the host reference using minimap2 v2.26-r1175 with the ONT preset (-ax map-ont). Alignments were processed with SAMtools v1.17 to retain only mapped primary alignments, excluding unmapped reads (flag 0 × 4), secondary alignments (flag 0 × 100), and supplementary/chimeric alignments (flag 0 × 800) (i.e., SAMtools view -F 2308). In addition, a CIGAR-based soft-clipping filter was applied to remove partial/unstable mappings: for each primary alignment, the fraction of soft-clipped bases (S) relative to the query length (computed from the CIGAR operations consuming the query: M/I/S/=/X) was calculated, and alignments with soft clipping > 15% were discarded. Read identifiers passing the host-mapping criteria were exported and used to remove the corresponding sequences from the original FASTQ, generating a final non-host FASTQ file for downstream metagenomic analysis. Intermediate files were retained for reproducibility (sorted BAM of host alignments, primary-only BAM, soft-clip-filtered BAM, and host read ID list). Because host depletion was intended to remove only confidently host-derived sequences, these conservative mapping criteria were adopted to minimize the inadvertent removal of viral reads while reducing host-derived background. The bioinformatics workflow for the virome analysis was entirely developed on the open-source Galaxy platform https://usegalaxy.org/ (accessed on 15 May 2026) [45]. The pipeline includes all preprocessing steps for Nanopore reads, such as adapter trimming and quality filtering. Although these operations can be performed directly by the MinKNOW v22.12.7 software, the pipeline was designed to accommodate datasets with varying levels of preprocessing (from none to fully processed), incorporating all necessary steps to ensure universal compatibility. The viral metagenomic analysis was performed using the taxonomic sequence classifier Kraken2 (Galaxy Version 2.1.1+galaxy1), which assigns taxonomic labels to sequencing reads [46], and employing the prebuilt database “Prebuilt Refseq indexes: Viral (Version: 2022-06-07—Downloaded: 2022-08-04T105935Z)” [47] for the taxonomic classification of viruses. Kraken2 was run using default taxonomic assignment parameters, which allow the classification of a read when at least one k-mer matches a reference sequence in the database. This configuration was adopted to maximize analytical sensitivity in a discovery-oriented metagenomic context. To increase the sensitivity of viral detection in an exploratory metagenomic context, downstream analyses were restricted to the family taxonomic level, considering also assignments supported by a single read. Similar approaches have been adopted in previous Oxford Nanopore-based metagenomic studies, in which even single reads were considered useful for indicating the potential presence of viral taxa [48,49,50]. The workflow used for the metagenomic analysis of the data is publicly available and can be downloaded and utilized at the following link (https://galaxy-main.usegalaxy.org/published/workflow?id=d5436cee11a34de9 accessed on 15 May 2026) [51]. Detailed information on the use of the workflow on the Galaxy platform is provided in the Supplementary Material. For each of the six sequenced samples, an Excel file was generated containing the results of the metagenomic analysis. All the processed results of the metagenomic analysis were grouped into a single file .xlsx (Dataset 7), available in the Figshare repository at https://doi.org/10.6084/m9.figshare.27909843 [37]. Although the Excel (.xlsx) files generated from the Kraken analysis facilitate the identification of read assignments at the species level, our analysis is limited to the family level. This decision is designed to reinforce the reliability of taxonomic assignments, given the inherent variability of broad-range metagenomic investigations and the relatively high error rate (approximately 6%) associated with Nanopore sequencing [52]. To ensure conceptual clarity, we specify below the operational meaning of the term relative abundance, which is calculated as the proportion of reads assigned to each viral taxon relative to the total number of classified viral reads, expressed as a percentage [53,54,55].

Finally, the analyses presented in this study were conducted using the Prebuilt RefSeq Viral database (version 2022-06-07, downloaded on 8 April 2022) [47], in which some viral taxa are classified according to nomenclature that does not fully reflect the current ICTV taxonomy. For example, viruses currently assigned to the order Reovirales are still classified within the family *Reoviridae*. Similarly, bacteriophage taxa assigned by the database to *Myoviridae*, *Siphoviridae*, and *Podoviridae* correspond to members of the class Caudoviricetes under the current ICTV taxonomy [56]. In this manuscript, taxonomic assignments are reported as returned by the reference database and, where relevant, interpreted according to the updated ICTV taxonomy. For terminological accuracy, the term viral taxa will thus be used throughout the manuscript.

## 3. Results and Discussion

The raw sequencing reads are deposited in the NCBI Sequence Read Archive under the unified BioProject PRJNA1199354. The same raw reads, together with FASTQ files containing the same reads after removal of host-derived sequences (host-depleted), are also available in the Figshare repository at https://doi.org/10.6084/m9.figshare.27909843 [37]. These data include, for all analyzed samples, raw sequencing reads in fastq.gz format and processed metagenomic analysis results in .xlsx format, reporting all information on read counts and relative abundances for each taxon. A detailed description of each dataset is provided in the Supplementary Material, and information on sequencing reads and virus-assigned reads, derived from the report generated by fastp before and after host depletion, is reported in Table S1 in the Supplementary Material.

### 3.1. Virome Composition in Aedes mariae and Breeding-Site Water

**Figure 1:**
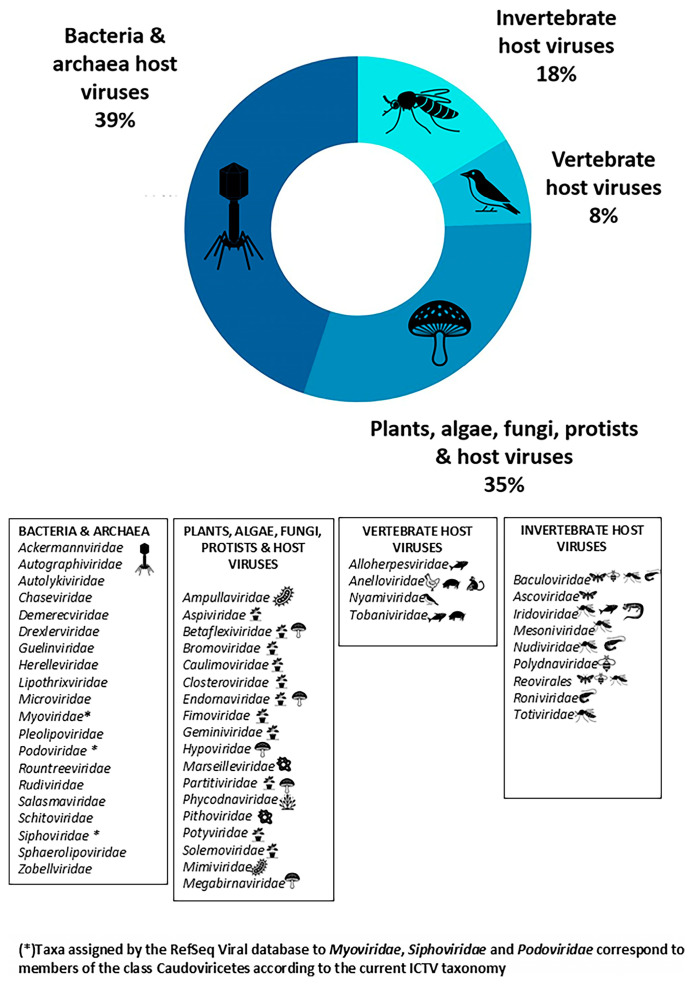
Viral taxa detected in rock pool water and across all mosquito life stages and sexes, grouped by host specificity. Taxa marked with an asterisk (*) are further explained in the note reported within the figure.

Metagenomic analysis of the virome associated with the breeding-site water and with mosquito individuals, after removing host-derived genomic sequences from the latter, identified a total of 51 viral taxa spanning multiple taxonomic ranks, including families, classes, and orders. These taxa were grouped according to host specificity, with taxonomic assignments interpreted according to the current ICTV taxonomy where applicable: bacteria and archaea (39%), viruses associated with plants, algae, fungi, and protists (35%), invertebrate-associated viruses (18%), and vertebrate-associated viruses (8%) (Figure 1).

The number of viral taxa varied across mosquito developmental stages and sexes, with 22 taxa in L4 larvae, 28 in female pupae, 26 in male pupae, 27 in female adults, and 31 in male adults. In the rock pool water sample, 20 viral taxa were identified (Dataset 7) [37].

**Figure 2:**
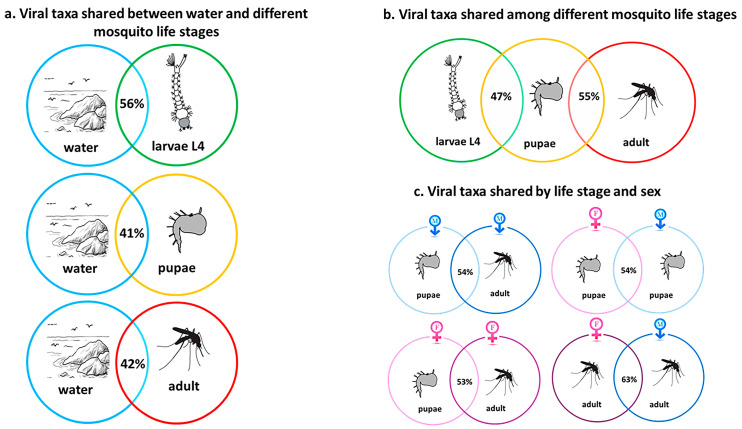
Overview of shared viral taxa in *Aedes mariae*, showing the percentage of shared viral taxa (**a**) between water and the different mosquito life stages, (**b**) among the different mosquito life stages, and (**c**) according to life stage and sex. The figure highlights patterns of viral occurrence and overlap across developmental transitions and between male and female mosquitoes. Percentages represent the overlap of viral taxa between the compared groups and were calculated as the ratio between the number of shared taxa and the total number of distinct viral taxa detected across the two groups considered.

The viral composition of the sea rock pool water partially overlaps with that of mosquito individuals at different developmental stages (Figure 2).

The life stage with the viral composition most similar to that of the pool water is the L4 larval stage, sharing 56% of the viral taxa, whereas the pupa and the adult share 41% and 42%, respectively. The remaining viral taxa were detected exclusively either in the breeding-site water or in the L4 larvae and were not shared between the two sample types (Figure 2a). When comparing viral taxa exclusively across mosquito developmental stages (Figure 2b), the L4 larval stage shares 47% of viral taxa with the pupa, whereas pupae and adults share 55% of the viral taxa. A comparison of virome similarity based on the same sex but at different life stages (Figure 2c) shows that the virome composition is 54% similar between male pupae and male adults, and 53% similar between female pupae and female adults. Comparing the same life stage but different sexes, the virome composition is 54% similar between female and male pupae, and 63% similar between female and male adults. Although some viral taxa were shared between the water and mosquito samples across developmental stages and sexes, other taxa were detected only in specific developmental stages within the analysed dataset. For instance, *Mesoniviridae* were detected exclusively in water, female pupae, and adult females. Similarly, *Pleolipoviridae* were observed only at the L4 larval stage, while *Tobaniviridae* and *Nyamiviridae* appeared to be restricted to adult mosquitoes of different sexes.

It is well established that the ecology of the breeding site significantly influences the microbiota composition of mosquitoes, as a large portion of the microbial community colonizing them is acquired during their aquatic life stage from larval habitats [9]. The larval stage may play an important role in the acquisition of environmental viruses, as larvae spend a prolonged period in water and actively feed by filtering the surrounding medium. However, further studies will be needed to determine the extent to which this process contributes to the assembly of the mosquito-associated virome. The lower proportion of shared taxa between larvae and pupae may be consistent with the extensive tissue reorganization that occurs during metamorphosis. Conversely, the higher similarity observed between pupae and adults may be compatible with the persistence of part of the virome through metamorphosis and after adult emergence. However, these interpretations remain speculative and were not directly assessed in the present study. This stage- and sex-associated distribution may suggest that, although a core set of viral taxa is shared throughout development, the relative contribution of individual taxa may vary depending on both the environment and the host stage, resulting in distinct virome compositions. Such heterogeneity could reflect selective pressures exerted by the host on its associated virome, although this hypothesis was not directly assessed in the present study. Moreover, the presence of certain viruses exclusively in mosquito individuals but not in water could be consistent with vertical transmission mechanisms, such as parent-to-offspring transfer. However, the absence of these viruses in water samples does not necessarily imply true absence, as they may occur at very low concentrations that remain undetected due to the lack of environmental replication and the resulting limited amplification of the viral signal in sequencing analyses. Given the exploratory design of the study, which was based on a single sampling site and a single sampling event without biological replication, the observations reported below should be interpreted as descriptive patterns within the analysed dataset rather than as generalizable ecological trends.

### 3.2. Shared Virome Composition and Abundance

**Figure 3:**
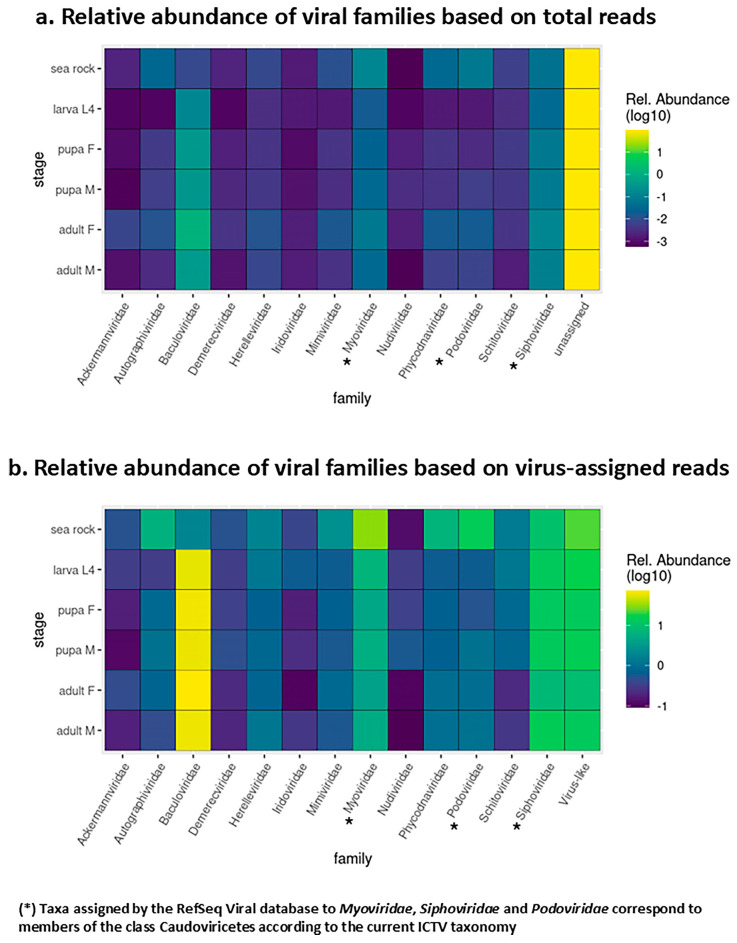
Heatmaps showing the relative abundance of viral families shared among water and mosquito samples across developmental stages. Values are displayed using a log10-transformed scale to facilitate the visualization of both highly abundant and low-abundance viral families. The color intensity reflects the relative abundance of viral families, with warmer colors (yellow) associated with higher values and cooler colors (dark purple) associated with lower values. (**a**) Relative abundance of viral families calculated as the proportion of reads assigned to each viral family relative to the total number of sequencing reads, (**b**) Relative abundance of viral families calculated as the proportion of reads assigned to each viral family relative to the total number of virus-assigned reads. Taxa marked with an asterisk (*) are further explained in the note reported within the figure.

Across all samples, 13 viral families were consistently shared between water and mosquito samples, accounting for 25% of the total taxa identified. Their relative abundance is shown in Figure 3 as heatmaps of log10-transformed percentages, highlighting differences in distribution across developmental stages when calculated relative to total sequencing reads (Figure 3a) or to virus-assigned reads (Figure 3b).

### 3.3. Description and Ecological Relevance of the Shared Virome

The shared virome was mainly composed of bacteriophages (69% of the detected taxa), including members of the families *Ackermannviridae*, *Autographiviridae*, *Demerecviridae*, *Herelleviridae*, and *Schitoviridae*, as well as Caudoviricetes taxa assigned by the RefSeq Viral database to the former families *Myoviridae*, *Siphoviridae* and *Podoviridae*, followed by insect-specific viruses (23%) belonging to *Baculoviridae*, *Nudiviridae*, and *Iridoviridae*, and by viruses associated with plants, algae, fungi, and protists (8%, *Phycodnaviridae*). Among them, in the *Aedes mariae* samples analysed, regardless of life stage or sex, *Baculoviridae* (0.14–1.3% of total reads and 55–73% of total assigned viral reads), Caudoviricetes taxa assigned to *Myoviridae* (0.02–0.06% and 4–7%) and *Siphoviridae* (0.034–0.139% and 8–15%) were the most prevalent viral groups across all mosquito life stages. In water from marine rock pools, the viral community was instead dominated by Caudoviricetes taxa assigned to *Myoviridae* (0.13% of total reads and 30% of total assigned viral reads), *Podoviridae* (0.067% and 15%), and *Siphoviridae* (0.046% and 10%), followed by *Phycodnaviridae* (0.031% and 7%), *Autographiviridae* (0.029% and 6%), *Mimiviridae* (0.011% and 2.5%), and *Baculoviridae* (0.008% and 2%). A large proportion of reads remained unassigned, indicating that a substantial fraction of the virome could not be classified using currently available reference databases.

The observed virome composition can be further interpreted in light of the known ecological and biological characteristics of the detected viral groups.

Bacteriophages may contribute to the regulation and diversification of holobiont-associated bacterial communities through ecological mechanisms such as the “Kill the Winner” model [23]. Experimental studies have also demonstrated that phages can selectively modulate mosquito-associated bacterial communities and influence mosquito development and fitness [57,58]. Because mosquito-associated microbiota can influence vector competence, phage-mediated changes in bacterial community composition may indirectly affect the replication and transmission of human pathogens, including dengue and Zika viruses [59]. Notably, several of the most abundant bacteriophage families detected in our dataset are commonly associated with aquatic environments characterized by high salinity, including Caudoviricetes taxa assigned by the RefSeq Viral database to *Siphoviridae* [60], *Myoviridae* [61], *Podoviridae* [62] and *Autographiviridae* [63]. These families are among the most abundant viral taxa in the water sample collected from the mosquito breeding site and are also part of the shared virome between water and mosquito individuals, further suggesting a possible ecological link between the breeding habitat and the mosquito-associated virome. The consistent detection of Caudoviricetes taxa assigned to *Myoviridae*, *Siphoviridae* and *Baculoviridae* in both mosquito samples and breeding-site water is consistent with a potential role of rock-pool water as a source of viral exposure during mosquito development. Together, these findings are consistent with the hypothesis that the aquatic environment may act as a reservoir and potential source of viral acquisition, highlighting the potential role of water in shaping and mediating viral transmission to the mosquito host.

Viruses infecting invertebrate hosts include Insect-specific viruses, including Mosquito-specific viruses, as well as viruses capable of infecting other invertebrate orders such as Crustacea, Lepidoptera, Coleoptera, and Orthoptera [64,65]. The Insect-specific viruses, including viral families *Baculoviridae*, *Nudiviridae*, *Mesoniviridae*, *Iridoviridae* and *Totiviridae*, are a group that primarily infects insects, although for some species there is evidence of possible transmission via arboviral pathways [66,67]. Insect-specific viruses are integral components of the mosquito holobiont and play key roles in host biology, evolution, and pathogen transmission [15,66,67,68]. For example, *Baculoviridae* include lethal pathogens for larvae of *Culex* and *Uranotaenia* [65,66,67] and can also infect adult *Culex nigripalpus*, *Culex quinquefasciatus* [64], and *Aedes triseriatus* larvae [69] (Federici & Lowe, 1972). However, recent evidence indicates that mosquito baculoviruses often exhibit limited pathogenicity and frequently establish persistent, subclinical, or latent infections rather than causing acute disease [65]. In particular, Chen et al. (2023) [70] describe mosquito baculoviruses (MBVs) as insect-specific DNA viruses with a narrow host range and a limited ability to cross the larval midgut barrier, resulting in infections that are often asymptomatic and may persist across developmental stages, potentially via vertical transmission. Baculoviruses can also adopt mixed transmission strategies, including both vertical and horizontal transmission, which facilitate viral persistence within host populations and allow reactivation under conditions of environmental or physiological stress [65]. Although baculoviruses can cause lethal infections in several insect groups, including lepidopterans, mosquitoes, and sawflies [71], and have therefore been developed as biological insecticides and biotechnological vectors [72,73], in most cases infections remain latent or sublethal, with the virus persisting or replicating at very low levels without causing overt mortality. Even in the absence of disease, such infections may influence host fitness and population dynamics [65]. *Nudiviridae* viruses have also been linked to mosquito infections [74], with the first mosquito-associated nudivirus identified in *Culex* and *Anopheles* by Feng et al. (2022) [75]. The *Mesoniviridae* family has been found in *Culex*, *Aedes*, *Anopheles*, and *Uranotaenia* [76], and includes newly discovered species such as *Cavally virus* and *Nam Dinh virus*, the latter capable of infecting vertebrates [77,78]. *Iridoviridae*, which infects both insects and some vertebrates, is mainly associated with *Aedes* and *Culex* [11], and can cause reduced fitness, mortality in larvae [79], and visible symptoms like iridescence [64], as well as a 20–35% reduction in progeny and shorter lifespan in adults [80]. We also detected viruses belonging to the order Reovirales, which includes families such as *Spinareoviridae* and *Sedoreoviridae*, characterized by a broad host range that includes arthropods. Within this order, insect-specific viruses are also present; among them, the genus *Dinovernavirus* comprises reoviruses associated with mosquitoes and has been isolated from species of the genus *Aedes* [81]. The *Totiviridae* family has been detected mainly in *Aedes* and *Anopheles* species [82,83]. Collectively, these families represent some of the most frequently observed viruses in mosquitoes [11]. Within Insect-specific viruses, *Mesoniviridae*, *Iridoviridae*, and *Totiviridae* are also considered Mosquito-specific viruses, whose diversity has expanded considerably in recent years, even though their role in vertebrate infections remains unclear. Taxonomic studies have revealed a close relationship between these viruses and arboviruses, suggesting that Mosquito-specific viruses may interfere with other viruses during co-infections [84].

With respect to viruses linked to plants and protists, they are likely acquired by mosquitoes during feeding on plant-derived sugars but do not replicate in the host [85,86]. Their role within the mosquito holobiont remains unclear.

Finally, vertebrate-associated viruses include taxa known to infect fish, amphibians [87], mammals [88], and birds [89]. No evidence currently supports replication or transmission of these viruses by mosquitoes. Although there is currently no evidence that *Aedes mariae* acts as a vector of pathogenic viruses, future studies incorporating species-level viral identification and phylogenetic analyses would help to better characterize the vertebrate-associated viral component detected in this study. Such analyses could clarify whether any of the detected viruses are evolutionarily related to known arboviruses or other pathogenic taxa and provide further insights into their ecological significance.

Although several viral families detected are commonly reported in other species of the genus *Aedes*, including *Flaviviridae*, *Phenuiviridae*, *Baculoviridae*, Rhabdoviridae, *Orthomyxoviridae*, *Picornaviridae*, *Reoviridae*, *Arenaviridae*, *Peribunyaviridae*, *Totiviridae*, *Astroviridae* and *Retroviridae* [90,91,92,93], additional families that constitute the *Aedes mariae* virome, such as *Mimiviridae*, and Caudoviricetes taxa assigned by the RefSeq Viral database to *Podoviridae*, *Myoviridae* and *Siphoviridae* [94], are typically associated with saline and marine environments. It is also noteworthy that previous studies [60,61] have shown that the families *Baculoviridae*, *Myoviridae* and *Siphoviridae*, which in our study were found to be the most abundant in the *Aedes mariae* virome, are among the most abundant viral families in high-salinity sites, further supporting the idea that the ecological characteristics of sea rock pools contribute to shaping the virome composition of this species.

### 3.4. Technical Issues

Another interesting finding that emerged from our study is that, prior to host genome removal, in mosquito individuals at all developmental stages, 96–98% of the reads assigned to viruses were classified within the *Baculoviridae* family (Dataset 9 in Supplementary Material) [37]. However, after host sequence filtering, the relative abundance of *Baculoviridae* showed a marked decrease, with a reduction of up to 40%. This indicates that in the dataset subjected to host depletion, the proportion of *Baculoviridae* is noticeably lower compared with the dataset prior to host removal. Nevertheless, *Baculoviridae* remains among the most abundant viral families in the analyzed samples. Considering the known error rate associated with Nanopore technology, as previously discussed, it cannot be excluded that a small fraction of host sequences may not be completely discriminated, and may therefore be slightly overestimated in the taxonomic assignments. This observation also suggests that a fraction of host-derived reads was initially misclassified as viral, indicating the presence of sequence homology between regions of the mosquito genome and baculoviral sequences. Such similarity may result from historical genetic interactions between viruses and their hosts. In particular, several studies have documented events of gene exchange or integration between viruses and insect genomes, leading to the presence of endogenous viral elements (EVEs) that retain similarity to contemporary viral taxa [95,96,97]. These elements are thought to derive from ancient viral infections that became integrated and subsequently fixed in host evolutionary lineages. Long-term virus–host coevolution can shape viral genome composition, as viruses often evolve nucleotide and codon usage patterns that resemble those of their hosts, reflecting shared evolutionary pressures between viral genomes and host coding regions [98], further increasing the likelihood that host-derived reads are initially classified as viral in metagenomic analyses. Consequently, the removal of host sequences can substantially reduce the apparent abundance of certain viral taxa, as observed in the present dataset for *Baculoviridae*.

Overall, the percentage of reads assigned to viruses was relatively low, as expected for untargeted metagenomic approaches. In addition, many reads remained unassigned, suggesting that a substantial fraction of the virome cannot be classified using the currently available reference databases and thus indicating the presence of a largely unexplored viral diversity. Given the exploratory nature of the present study and the still limited representation of viral diversity in current reference databases, the taxonomic assignment strategy was designed to maximize analytical sensitivity and facilitate the detection of potentially rare viral taxa. Consequently, no minimum read-count threshold was applied for taxon detection. However, taxonomic assignments supported by a low number of reads should be interpreted with caution, as metagenomic classification based on individual reads may be affected by sequencing errors, database incompleteness, or spurious matches. To mitigate the risk of overinterpretation, taxonomic inferences in the present study were primarily discussed at the family level, and low-abundance detections were considered indicative of the potential presence of a viral taxon rather than definitive evidence of its occurrence. Similar sensitivity-oriented approaches have been adopted in previous Oxford Nanopore-based metagenomic studies, where taxonomic assignments supported by very few reads, including single-read detections, were retained to maximize viral discovery potential [48,49,50]. Overall, although this methodological framework may represent a limitation of the present study, it reflects a deliberate trade-off between sensitivity and taxonomic certainty, consistent with the study’s primary discovery-oriented objective.

## 4. Conclusions

This study provides the first description of the virome associated with *Aedes mariae* and its sea-rock pool breeding habitat, offering the opportunity to explore how the distinctive ecological features of rock-pool water, such as high salinity and rapidly fluctuating environmental conditions, may influence the composition of the mosquito-associated virome. Our results are consistent with the hypothesis that the aquatic environment may represent a source of viral acquisition, with a subset of viral taxa being detected across multiple mosquito developmental stages and sexes. The dominance of bacteriophages and Insect-specific viruses is consistent with their potential role in the mosquito holobiont, as suggested by previous studies. Future analyses aimed at achieving finer taxonomic resolution will require more stringent classification parameters and the use of high-accuracy long-read sequencing technologies (e.g., Phred ≥ Q30). Given the exploratory nature of the study, future investigations including additional sites and sampling periods will help further elucidate the ecological processes underlying the patterns observed in the present dataset. Overall, this dataset expands current knowledge of mosquito virome and provides a valuable resource for future ecological and virological research on understudied mosquito species and extreme breeding habitats.

## Data Availability

The sequencing reads generated in this study are deposited in the NCBI Sequence Read Archive under the unified BioProject accession number PRJNA1199354. All datasets, including raw sequencing reads in .fastq.gz file compressed in gzip format and processed metagenomic results in .xlsx format, are also available in the Figshare repository at https://doi.org/10.6084/m9.figshare.27909843. The shared files include single-end reads from *Aedes mariae* specimens (larvae, pupae, and adults) and water samples from sea-rock pools, generated using the long-read sequencing technology from Oxford Nanopore Technologies (ONT), as well as two spreadsheets summarizing virome composition and relative abundance.

## Supplementary Materials

The following supporting information can be downloaded at: https://www.mdpi.com/article/10.3390/biology15120940/s1. Figure S1 Workflow of the virome characterization pipeline and Table S1 Summary of sample processing and viral read assignment results generated by the fastp tool, before and after host genome removal.
