# Supplementary material for: Exploratory Metaviromic Analysis of the Sea-Rock Pool Mosquito Aedes mariae and the Water of Its Breeding Habitat
Source: Biology (Basel). 2026 Jun 16;15(12):940. doi: 10.3390/biology15120940 (PMC13295856; doi:10.3390/biology15120940)
Supplement: Supplementary file 1 [file biology-15-00940-s001.zip › biology-4357118-supplementary.pdf]

Supplementary materials

Library preparation, sequencing and bioinformatic analysis

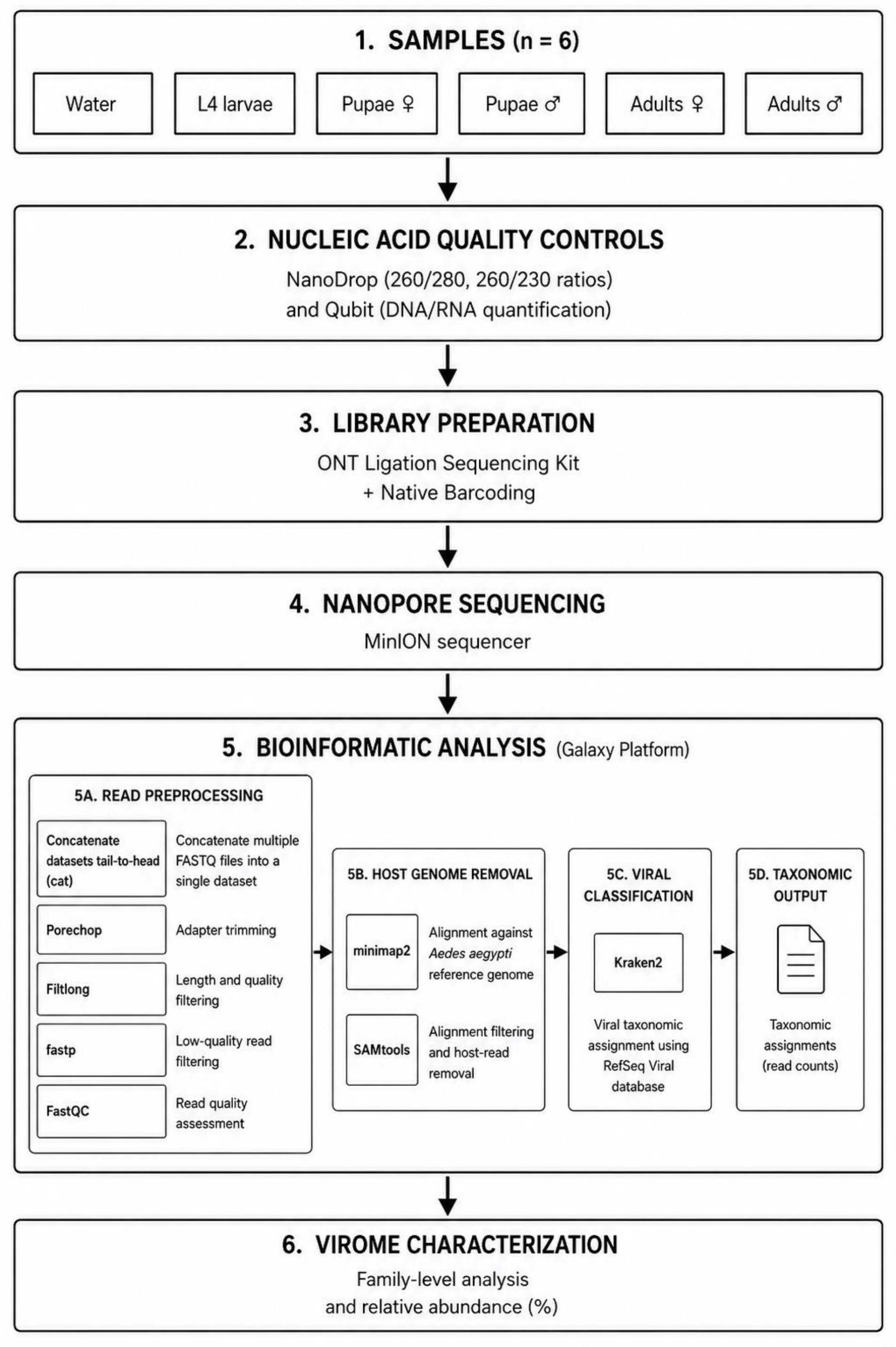

Figure S1 Workflow of the virome characterization pipeline

To use our workflow on the web-based open-source platform Galaxy, you need to register by creating an account at the following link: https://usegalaxy.org/. You can utilize our workflow directly on your raw reads output from MinION sequencing. The steps to follow are:

1. Create a new project in the “History” section.

2. Upload your raw reads files from the sequencing output that still need concatenation.

3. **Download our workflow** in **.ga format** from the following link: https://galaxy-main.usegalaxy.org/published/workflow?id=d5436cee11a34de9**.** Then, go to the **“Workflows”** section on the left-hand menu, click on **“Import workflow”**, and import our workflow in **.ga format**. Alternatively, you can search for our workflow by going to the **“Workflow”** section, then **“Public Workflow”**, and searching for **“Workflow_Metagenomic_Virus_Oxford Nanopore Technology”**. Click on **“Run”** to execute it.

We also recommend reviewing the tutorials available in the “See Galaxy Training Materials” section on the platform's website.

Results

Data Records

The sequencing reads are stored in the NCBI Sequence Read Archive and organized under the unified BioProject PRJNA1199354 and are available, along with the rest of the datasets, in the Figshare repository at https://doi.org/10.6084/m9.figshare.27909843. These data include raw sequencing reads in fastq.gz format and processed metagenomic analysis results in .xlsx format.

Dataset 1- sea rock pool water_barcode04.fastq.gz

The file contains concatenated raw single-end sequencing reads, provided as a single file in *.fastq.gz* format, compressed in gzip format, derived from the "Sea rock pool water" sample. The raw reads were generated using the long-read sequencing technology from Oxford Nanopore Technologies (ONT) with Ligation Sequencing Kit 9 (SQK-LSK109) and Native Barcoding Expansions (EXP-NBD104 and EXP-NBD114) on FLO-MIN106 (R9.4.1) flow cells using the MinION Mk1B sequencer. The FASTQ file has a total size of 385.95 MB.

Dataset 2- ID 4_larva L4_barcode01.fastq.gz

The file contains concatenated raw single-end sequencing reads, provided as a single file in *.fastq.gz* format, compressed in gzip format, derived from the "larva L4" sample. The raw reads were generated using the long-read sequencing technology from Oxford Nanopore Technologies (ONT) with Ligation Sequencing Kit 9 (SQK-LSK109) and Native Barcoding Expansions (EXP-NBD104 and

EXP-NBD114) on FLO-MIN106 (R9.4.1) flow cells using the MinION Mk1B sequencer. The FASTQ file has a total size of 355.29 MB

Dataset2_ID4_larvaL4_barcode01.non_host.fastq.gz

The file contains concatenated raw single-end sequencing reads, provided as a single file in *.fastq.gz* format, compressed in gzip format, derived from the "larva L4" sample. The raw reads were generated using the long-read sequencing technology from Oxford Nanopore Technologies (ONT) with Ligation Sequencing Kit 9 (SQK-LSK109) and Native Barcoding Expansions (EXP-NBD104 and EXP-NBD114) on FLO-MIN106 (R9.4.1) flow cells using the MinION Mk1B sequencer. The FASTQ file has a total size of 260.74 MB. Host-derived reads were removed by alignment to a mosquito reference genome, as described in the Materials and Methods section.

Dataset 3- ID 9_pupa F_barcode05.fastq.gz

The file contains concatenated raw single-end sequencing reads, provided as a single file in *.fastq.gz* format, compressed in gzip format, derived from the "pupa Female" sample. The raw reads were generated using the long-read sequencing technology from Oxford Nanopore Technologies (ONT) with Ligation Sequencing Kit 9 (SQK-LSK109) and Native Barcoding Expansions (EXP-NBD104 and EXP-NBD114) on FLO-MIN106 (R9.4.1) flow cells using the MinION Mk1B sequencer. The FASTQ file has a total size of 433.05 MB.

Dataset3_ID9_pupaF_barcode05.non_host.fastq.gz

The file contains concatenated raw single-end sequencing reads, provided as a single file in *.fastq.gz* format, compressed in gzip format, derived from the "pupa Female" sample. The raw reads were generated using the long-read sequencing technology from Oxford Nanopore Technologies (ONT) with Ligation Sequencing Kit 9 (SQK-LSK109) and Native Barcoding Expansions (EXP-NBD104 and EXP-NBD114) on FLO-MIN106 (R9.4.1) flow cells using the MinION Mk1B sequencer. The FASTQ file has a total size of 312.07 MB. Host-derived reads were removed by alignment to a mosquito reference genome, as described in the Materials and Methods section.

Dataset 4- ID 12_pupa M_barcode08.fastq.gz

The file contains concatenated raw single-end sequencing reads, provided as a single file in *.fastq.gz* format, compressed in gzip format, derived from the "pupa Male" sample. The raw reads were generated using the long-read sequencing technology from Oxford Nanopore Technologies (ONT) with Ligation Sequencing Kit 9 (SQK-LSK109) and Native Barcoding Expansions (EXP-NBD104 and EXP-NBD114) on FLO-MIN106 (R9.4.1) flow cells using the MinION Mk1B sequencer. The FASTQ file has a total size of 660.47 MB.

Dataset4_ID12_pupaM_barcode08.non_host.fastq.gz

The file contains concatenated raw single-end sequencing reads, provided as a single file in *.fastq.gz* format, compressed in gzip format, derived from the "pupa Male" sample. The raw reads were generated using the long-read sequencing technology from Oxford Nanopore Technologies (ONT) with Ligation Sequencing Kit 9 (SQK-LSK109) and Native Barcoding Expansions (EXP-NBD104

and EXP-NBD114) on FLO-MIN106 (R9.4.1) flow cells using the MinION Mk1B sequencer. The FASTQ file has a total size of 478.97 MB. Host-derived reads were removed by alignment to a mosquito reference genome, as described in the Materials and Methods section.

Dataset 5- ID 15_adult F_barcode11.fastq.gz

The file contains concatenated raw single-end sequencing reads, provided as a single file in *.fastq.gz* format, compressed in gzip format, derived from the "adult Female" sample. The raw reads were generated using the long-read sequencing technology from Oxford Nanopore Technologies (ONT) with Ligation Sequencing Kit 9 (SQK-LSK109) and Native Barcoding Expansions (EXP-NBD104 and EXP-NBD114) on FLO-MIN106 (R9.4.1) flow cells using the MinION Mk1B sequencer. The FASTQ file has a total size of 356.76 MB

Dataset5_ID15_adultF_barcode11.non_host.fastq.gz
The file contains concatenated raw single-end sequencing reads, provided as a single file in *.fastq.gz* format, compressed in gzip format, derived from the "adult Female" sample. The raw reads were generated using the long-read sequencing technology from Oxford Nanopore Technologies (ONT) with Ligation Sequencing Kit 9 (SQK-LSK109) and Native Barcoding Expansions (EXP-NBD104 and EXP-NBD114) on FLO-MIN106 (R9.4.1) flow cells using the MinION Mk1B sequencer. The FASTQ file has a total size of 247.84 MB. Host-derived reads were removed by alignment to a mosquito reference genome, as described in the Materials and Methods section.

Dataset 6- ID 19_adult M_barcode18.fastq.gz

The file contains concatenated raw single-end sequencing reads, provided as a single file in *.fastq.gz* format, compressed in gzip format, derived from the "adult Male" sample. The raw reads were generated using the long-read sequencing technology from Oxford Nanopore Technologies (ONT) with Ligation Sequencing Kit 9 (SQK-LSK109) and Native Barcoding Expansions (EXP-NBD104 and EXP-NBD114) on FLO-MIN106 (R9.4.1) flow cells using the MinION Mk1B sequencer. The FASTQ file has a total size of 3.10 GB.

Dataset6_ID19_adultM_barcode18.non_host.fastq.gz
The file contains concatenated raw single-end sequencing reads, provided as a single file in *.fastq.gz* format, compressed in gzip format, derived from the "adult Male" sample. The raw reads were generated using the long-read sequencing technology from Oxford Nanopore Technologies (ONT) with Ligation Sequencing Kit 9 (SQK-LSK109) and Native Barcoding Expansions (EXP-NBD104 and EXP-NBD114) on FLO-MIN106 (R9.4.1) flow cells using the MinION Mk1B sequencer. The FASTQ file has a total size of 2.43 GB. Host-derived reads were removed by alignment to a mosquito reference genome, as described in the Materials and Methods section.

Dataset 7- Report metagenomic analyses.xlsx

The file contains two tables summarizing the results of the metagenomic analyses of the *Aedes mariae* virome across the six samples. The first table reports information on reads before host sequence

removal (host depletion), while the second table reports the information after host depletion. The file is in .xlsx format.

Dataset 8 - Shared virome – composition and relative abundances.xlsx

The file contains two tables reporting information associated with the 13 viral families composing the virome shared between water and mosquito developmental stages, together with the **Virus-like** category. The first table (**total number of virus-assigned reads**) reports the number of reads assigned to each viral family (or Virus-like category) and their corresponding relative abundances calculated with respect to the total number of virus-assigned reads. The second table (**total number of reads**) reports the same viral categories together with unassigned reads, and relative abundances are calculated with respect to the total number of sequencing reads remaining after host depletion. The **Virus-like** category includes reads classified by the Kraken2 reference database as showing significant similarity to viral reference sequences but lacking sufficient taxonomic resolution for reliable assignment to a recognized viral family, genus, or species. All reads considered correspond to the dataset obtained after host depletion.

Dataset 9 - Baculoviridae_Mosquitoes_RelAb_PrePostHostDep

The file contains tables reporting the number of reads assigned to viruses belonging to the family *Baculoviridae* detected in mosquito samples. The .xlsx file reports the number of reads assigned to each viral taxon and their corresponding relative abundance values. Relative abundances are calculated with respect to the total number of virus-assigned reads. Values are reported for samples before and after host depletion.

Table S1 Summary of sample processing and viral read assignment results generated by the fastp tool, before and after host genome removal

	**Dataset 1**	**Dataset 2**	**Dataset 3**	**Dataset 4**	**Dataset 5**	**Dataset 6**
**Sample type**	rock pool water	*Ae. mariae* L4 larvae	*Ae. mariae* female pupae	*Ae. mariae* male pupae	*Ae. mariae* adult females	*Ae. mariae* adult males
**Total filtered reads before host depletion (K)**	175.525	154.965	135.770	230.266	112.449	1111.782
**Total filtered reads after host depletion (K)**	n.a.	128.399	104.931	162.272	53.229	856.753
**Sequence length (bp)**	400-41904	400-33352	400-42954	400-35209	400-49127	400-49562
**Mean read length after filtering, before host depletion (bp)**	1305	1697	2271	2285	2830	2477

**Mean read length after filtering, after host depletion (bp)**	n.a.	1707	2431	2714	4579	2837
**N° total reads assigned to viruses before host depletion**	725	2526	4200	9337	10.611	44.238
**N° total reads assigned to viruses after host depletion**	n.d.	278	514	760	864	4844
